# Complete mitochondrial genomes of the human follicle mites *Demodex brevis* and *D. folliculorum*: novel gene arrangement, truncated tRNA genes, and ancient divergence between species

**DOI:** 10.1186/1471-2164-15-1124

**Published:** 2014-12-16

**Authors:** Michael F Palopoli, Samuel Minot, Dorothy Pei, Alicia Satterly, Julie Endrizzi

**Affiliations:** Department of Biology, Bowdoin College, Brunswick 6500, College Station, ME 04011 USA

**Keywords:** Chelicerata, Acari, Acariformes, *Demodex*, Follicle mites, Mitochondria, Genome rearrangements, tRNA structure, Coevolution

## Abstract

**Background:**

Follicle mites of the genus *Demodex* are found on a wide diversity of mammals, including humans; surprisingly little is known, however, about the evolution of this association. Additional sequence information promises to facilitate studies of *Demodex* variation within and between host species. Here we report the complete mitochondrial genome sequences of two species of *Demodex* known to live on humans—*Demodex brevis* and *D. folliculorum*—which are the first such genomes available for any member of the genus. We analyzed these sequences to gain insight into the evolution of mitochondrial genomes within the Acariformes. We also used relaxed molecular clock analyses, based on alignments of mitochondrial proteins, to estimate the time of divergence between these two species.

**Results:**

Both *Demodex* genomes shared a novel gene order that differs substantially from the ancestral chelicerate pattern, with transfer RNA (tRNA) genes apparently having moved much more often than other genes. Mitochondrial tRNA genes of both species were unusually short, with most of them unable to encode tRNAs that could fold into the canonical cloverleaf structure; indeed, several examples lacked both D- and T-arms. Finally, the high level of sequence divergence observed between these species suggests that these two lineages last shared a common ancestor no more recently than about 87 mya.

**Conclusions:**

Among Acariformes, rearrangements involving tRNA genes tend to occur much more often than those involving other genes. The truncated tRNA genes observed in both *Demodex* species would seem to require the evolution of extensive tRNA editing capabilities and/or coevolved interacting factors. The molecular machinery necessary for these unusual tRNAs to function might provide an avenue for developing treatments of skin disorders caused by *Demodex*. The deep divergence time estimated between these two species sets a lower bound on the time that *Demodex* have been coevolving with their mammalian hosts, and supports the hypothesis that there was an early split within the genus *Demodex* into species that dwell in different skin microhabitats.

**Electronic supplementary material:**

The online version of this article (doi:10.1186/1471-2164-15-1124) contains supplementary material, which is available to authorized users.

## Background

**Figure 1:**
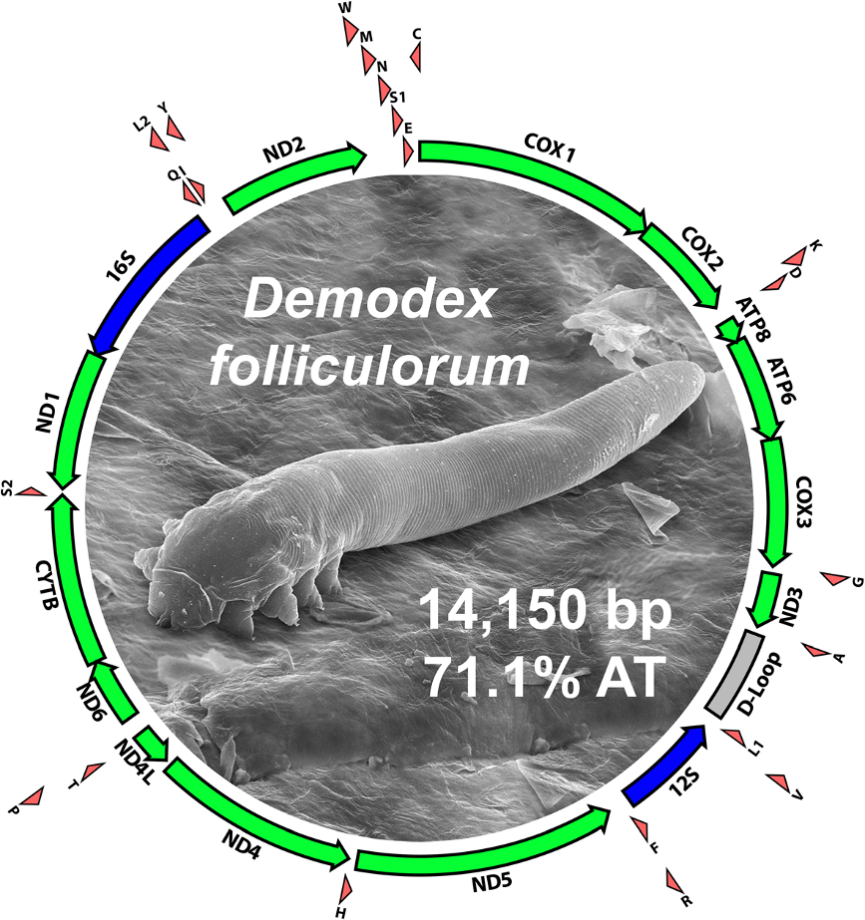
**Organization of the mitochondrial genome of** ***Demodex folliculorum*** **.** Protein-coding, ribosomal RNA, and transfer RNA genes are depicted as green, blue, and red arrows, respectively. The arrows represent the direction of transcription. The AT-rich, putative control region is depicted as a gray box (labeled “D-Loop”). Inside the circle is a scanning electron micrograph of an individual *D. folliculorum* (image credit: Power and Syred). The identical gene arrangement was observed in the *D. brevis* mitochondrial genome. This is a novel gene arrangement among the acariform mitochondrial genomes sequenced to date. Like other members of the Acariformes, *Demodex* proved to have a compact and AT-rich mitochondrial genome.

Mites of the genus *Demodex* live in the hair follicles and sebaceous glands of mammalian skin [1]. They are extremely widespread among mammalian lineages, with species having been described from hosts in three of the seven marsupial orders, and in 11 of the 18 eutherian orders [2]. Their bodies exhibit specializations that make them well adapted to inhabiting the constricted spaces of the pilosebaceous complex—they are typically just 100–300 μM long and cylindrical in shape, with extremely reduced legs and setation (Figure 1). Together, these observations suggest an ancient, coevolutionary relationship between mammals and the *Demodex* species that inhabit their skin; yet surprisingly little is known about the evolutionary history or dynamics of this association.

*Demodex* are ubiquitous in humans and thought to contribute to medically important skin disorders. Both recent molecular studies [3], and studies in which the skin of cadavers was sampled extensively (reviewed in [2]), suggest that the incidence of infestation approaches 100%. Despite the high incidence of *Demodex* itself, the frequency with which *Demodex* causes pathogenicity appears to be very low, which has led to their characterization as human commensals by many authors. Nevertheless, there are reports of high *Demodex* densities associated with two medically important disorders of the skin—marginal blepharitis [4] and acne rosacea [5]. Furthermore, mite densities are known to increase and cause skin diseases when the immune system is compromised [6]. Treatment with acaricides, such as ivermectin, can be curative for some of these diseases [7]. These results suggest that *Demodex* should be considered parasitic in some host individuals. Future studies examining the potential roles of *Demodex* in skin disorders, as well as interactions between *Demodex* and the host immune system, would be aided by an increase in the genetic markers available for distinguishing mite populations.

More generally, greater genetic information would facilitate the study of *Demodex* variation within and between host species. For example, mite genetic variation could be investigated within a host species in order to better understand the dynamics of movement among host individuals [8], or even to provide information about the migration patterns of the hosts themselves [9]. Similarly, the question of how often these mites migrate across host species boundaries could be addressed by making comparisons of host and mite molecular phylogenies [10].

Two species of *Demodex* inhabit the skin of humans, with histological studies suggesting that each occupies a different niche: *Demodex folliculorum* typically resides in the hair follicle nearer the skin surface, whereas *D. brevis* is generally found deeper in the sebaceous glands [1]. Although distinguished initially based on morphological differences, recent molecular work using both mitochondrial and nuclear gene markers has verified that these are distinct species [11, 12]. Indeed, the observed levels of sequence divergence suggest that they are not closely related species, although no estimate of divergence time is currently available.

Complete mitochondrial genomes have been determined for 685 arthropods; just 15 of these sequences are from the Acariformes, however, despite the more than 41,000 described species, and perhaps many times more undescribed species, within this division of the Acari [13]. The paucity of mitochondrial genomes available from among the Acariformes is especially unfortunate given their tendency to evolve unusual gene arrangements and truncated examples of tRNA genes [14–19]. To improve our understanding of the evolution of these features, additional examples of mitochondrial genomes from this important group of organisms are needed.

Here we report the complete mitochondrial genomes of both *D. brevis* and *D. folliculorum*, the first available for any species within the genus *Demodex*. Both species shared a novel mitochondrial gene order that has diverged extensively from the ancestral chelicerate pattern, with tRNA genes apparently having moved much more often than other genes. Furthermore, the mitochondrial genomes of both *Demodex* species possessed extremely truncated tRNA genes, with several of these lacking the sequence necessary to code for both D- and T-arms. Finally, we estimated the divergence time between the two human-associated *Demodex* species by using the sequences of mitochondrial proteins to conduct relaxed molecular clock analyses among Acariformes.

## Results and Discussion

### Mitochondrial genome content and organization

Like other members of the Acariformes, both species of *Demodex* had compact and AT-rich mitochondrial genomes. The *D. brevis* genome [GenBank accession number: KM114225] length was 14,211 bp and AT-percentage was 69.0%; the *D. folliculorum* genome [Genbank accession number: KM114226] length was 14,150 bp and AT-percentage was 71.1%. For comparison, the 15 other species of Acariformes with complete mitochondrial genomes available in GenBank exhibited an average genome size of 14,240 bp (SE 281 bp) and an average AT-percentage of 74.9% (SE 1.7%). These values agree closely with those that we observed for both *Demodex* species, suggesting that the same combinations of mutation pressures and selective forces that determine these parameters for other Acariformes are also operating in the *Demodex* lineage.

Most metazoans have mitochondrial genomes that are at least several hundred base pairs larger than those of the Acariformes [20]. Having a small mitochondrial genome would seem to have the obvious benefit of being less costly and time-consuming to replicate. Nevertheless, it remains unclear why selection for reduced genome size would be especially effective in the Acariformes. One of the contributing factors, however, is the extreme truncation of mitochondrial tRNA genes that is widespread in these organisms (see below).

High AT content is a common feature of metazoan mitochondrial genomes [21]. It has been hypothesized that this bias results from being housed where there are high concentrations of reactive oxygen species that promote GC to AT mutations [22]. Alternatively, AT-richness could be an adaptation for metabolic efficiency, with the higher energy cost and more limited availability of G and C nucleotides driving this pattern [23].

Mitochondrial genomes of both *Demodex* species possessed the standard collection of 13 protein-coding, two ribosomal RNA (rRNA), and 22 tRNA genes (Figure 1) present in the mitochondrial genomes of most metazoans [24]. The arrangement of these genes was identical in the two *Demodex* species, but novel among Acariformes. The tendency to accumulate rearrangements of the mitochondrial genome appears to be a common feature among the Acariformes [14–19]. Whether the accumulation of rearrangements in this taxonomic group is due to a higher structural mutation rate, or to relaxed natural selection on gene order, remains an open question.

**Figure 2:**
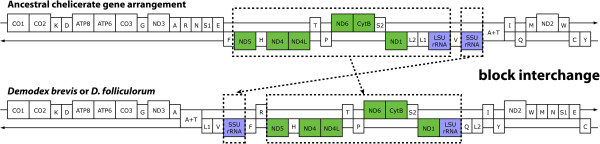
**Evolution of the** ***Demodex*** **gene arrangement from the ancestral chelicerate.** Circular mitochondrial genomes are depicted as linearized, starting at the 5′ end of the COX1 gene. The one letter amino acid code is used to designate the tRNA genes, with the exceptions that L1 = CUN; L2 = UUR; S1 = AGN; and S2 = UCN. The putative control region is designated as A + T to indicate that this has a higher AT% than the rest of the genome. Genes above the medial line are encoded on one strand, while those below the line are encoded on the other strand. As shown, the arrangement of protein-coding plus rRNA genes can be derived via one block interchange, with a minimum of three breakpoints. This could have occurred via a tandem duplication followed by differential loss of genes between the two tandem copies. Alternatively, it could have resulted from direct translocation, either of the 12S gene or of the interval that stretches from ND5 to the 16S gene. The tRNA genes seem to have moved independently of the other genes, since it would require a minimum of 15 breakpoints to explain the evolution of the *Demodex* arrangement if tRNA genes are included in the analysis.

The ancestral chelicerate gene arrangement, one shared by members of at least five orders, is exemplified by *Limulus polyphemus* [25, 26]. If we consider just the protein-coding and rRNA genes, the gene arrangement shared by both *Demodex* species can be derived from the ancestral chelicerate gene arrangement via a single block interchange (Figure 2), requiring three breakpoints (one breakpoint denotes when the linear sequence of genes is broken and rearranged such that what were two adjacent genes in the original genome no longer appear consecutively in the rearranged genome). Mechanistically, this block interchange could have resulted from a tandem duplication of the entire interval, followed by differential loss of functional genes in the two tandem copies. Alternatively, it could have resulted from direct translocation, either of the 12S gene or of the interval that stretches from ND5 to the 16S gene.

If tRNA genes are also considered in the analysis, however, the extent of rearrangement necessary to derive the *Demodex* mitochondrial gene order from the chelicerate ancestral pattern increases dramatically, requiring a minimum of 15 breakpoints. If tRNA genes usually move together with the other genes, then the estimated number of breakpoints should have been similar in both analyses. Hence, this increase in the minimum number of breakpoints necessary suggests that mitochondrial tRNA genes have a tendency to move more readily than protein-coding and rRNA genes. Evidence that tRNA genes tend to be more mobile than other genes within the mitochondria of some metazoan groups has been reported previously [20, 27, 28]. One plausible hypothesis is that this rapid movement is simply a consequence of their smaller size—structural rearrangements involving smaller elements may just happen to be more likely. For example, all other things being equal, the probability of a mitochondrial gene rearrangement causing a selective disadvantage may be greater for a larger gene than it is for a smaller gene, simply because the chances are greater that the rearrangement will encompass the entire gene. Alternatively, tRNA genes may be more mobile because they tend to move via a different, and unknown, mechanism.

**Figure 3:**
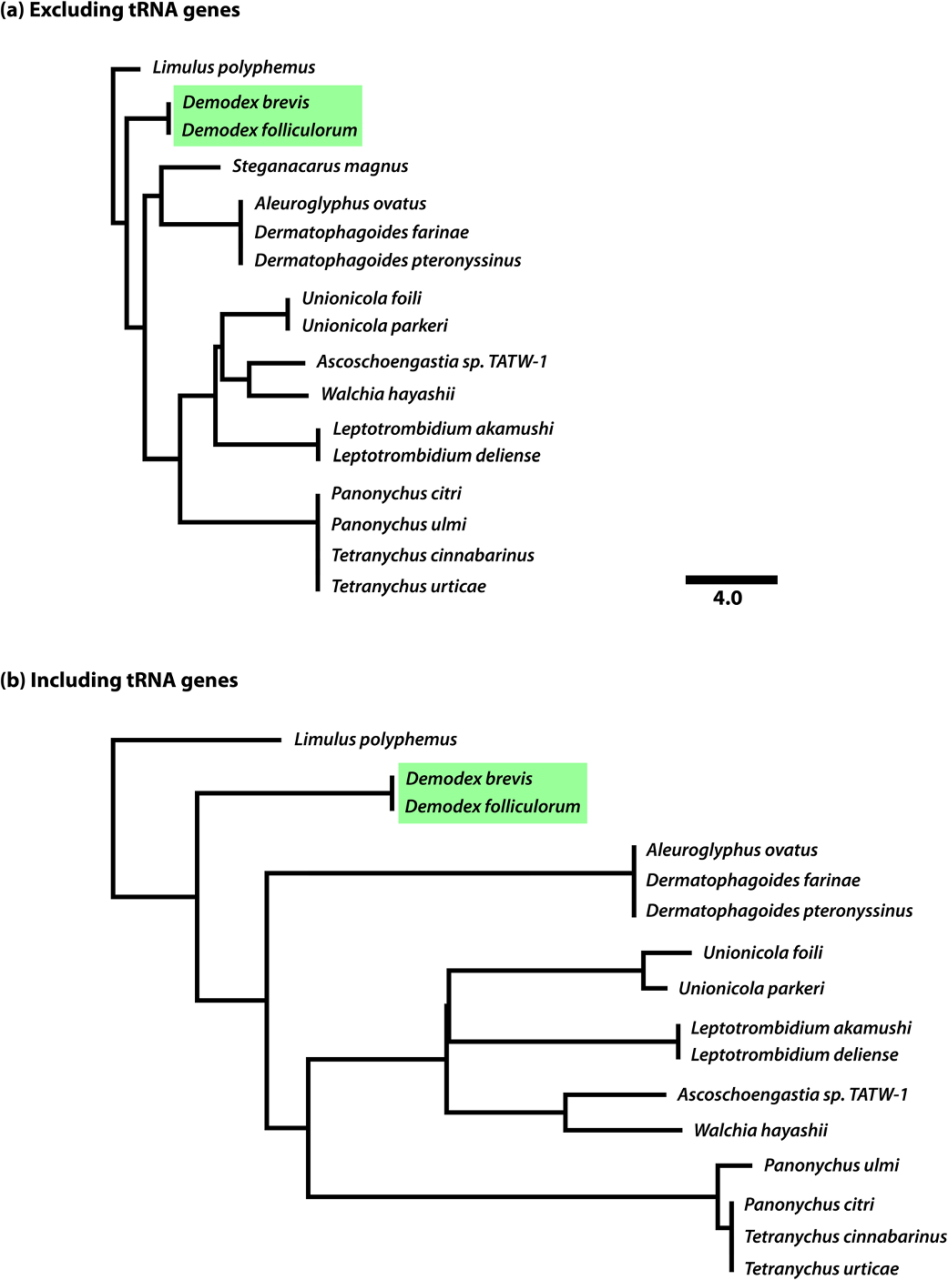
**Tree showing the breakpoint distances between gene arrangements.** Distances on this tree represent the minimum numbers of breakpoints necessary to transform one gene arrangement into another for all pairwise species comparisons. *Limulus polyphemus* (the horseshoe crab) is considered to represent the ancestral gene arrangement, since it has a gene order that is shared broadly across the chelicerates. In panel **(a)**, only the arrangements of protein-coding and rRNA genes were considered. In panel **(b)**, all mitochondrial genes were considered, including tRNA genes. Both trees are drawn to the same scale. All of the Acariformes have gene arrangements that have diverged substantially from that of *L. polyphemus*, and the tRNA genes have experienced substantially more rearrangements than the protein-coding or rRNA genes in all lineages. Both *Demodex* species shared an arrangement that is closer to the chelicerate ancestral arrangement than any other acariform species sequenced to date.

To compare the degree of rearrangement among Acariformes, and to determine whether a similar trend towards greater movement of tRNA genes holds throughout this group, we determined the minimum number of breakpoints necessary to derive each available species’ gene sequence from that of every other species. The results can be depicted as a breakpoint distance tree, in which the number of breakpoints that separates two genome sequences is represented as a distance. We conducted this analysis separately for just the protein-coding and rRNA genes (Figure 3a), and for all of the mitochondrial genes together, including the tRNA genes (Figure 3b). For all lineages, the number of breakpoints necessary to derive the gene order from the ancestral arrangement went up substantially when tRNA genes were included. This result indicates that the more rapid movement of tRNA genes is a general feature of mitochondrial genomes in the Acariformes.

Regardless of whether tRNA genes are included in the analysis, *Demodex* exhibited a lower degree of rearrangement than has been observed in any other member of the Acariformes to date (Figure 3). In other words, the *Demodex* arrangement was more similar to the ancestral chelicerate gene arrangement, whereas taxa such as *Leptotrombidium* or *Tetranychus* have apparently experienced many more rearrangements*.* It remains unclear whether different lineages within the Acariformes actually differ in the likelihood that they will experience rearrangements within a given period of time, or if this is simply the amount of variation among lineages that we should expect to see when considering the accumulation of low-frequency events. In support of the latter hypothesis, some taxa in different genera (i.e., *Panonychus* versus *Tetranychus*), or even different families (i.e., *Aleuroglyphus* versus *Dermatophagoides*), share identical gene arrangements, which suggests that the observed changes in gene order must happen rarely.

The putative control regions were identified in both species based on the following features: (1) they were by far the largest stretches of apparently noncoding DNA (length of 587 bp in *D. brevis*, 563 bp in *D. folliculorum*); (2) they had higher than average AT% (81.7% in *D. brevis*, and 75.7% in *D. folliculorum*); (3) they were in a conserved position in both species; and (4) the sequences of these regions have apparently been evolving more quickly than the rest of the mitochondrial genome, since they are so divergent that most of the segment could not even be aligned between the two species. All of these features are common observations of the control region in metazoan mitochondrial genomes [29]. Furthermore, the almost complete lack of similarity observed between control region sequences suggests that these lineages split a long time ago.

### tRNA gene structures

**Figure 4:**
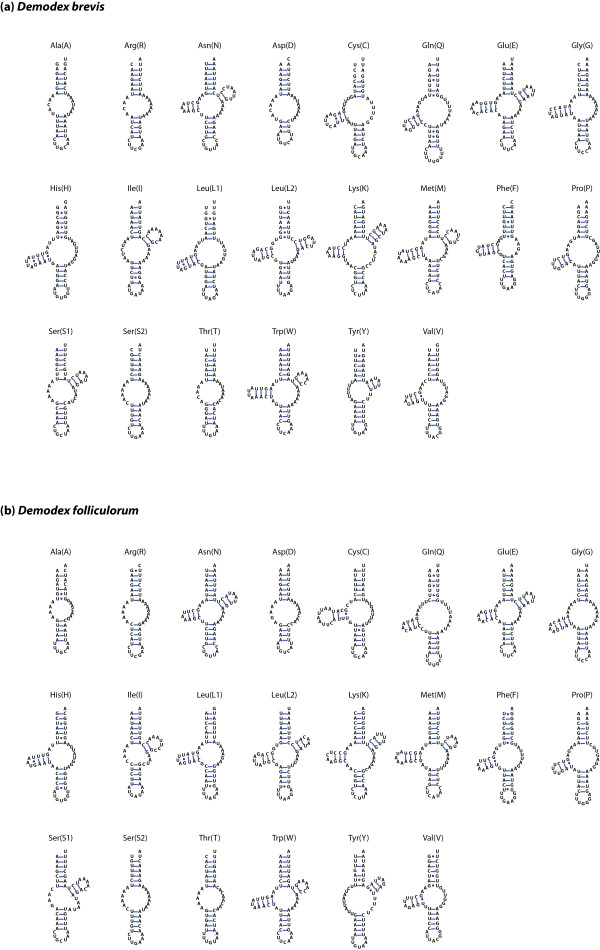
**Inferred structures of the mitochondrial tRNAs in both** ***Demodex*** **species.** Structures are arranged in alphabetical order. Each tRNA gene is named according to the one-letter amino acid abbreviation, except that L1 = CUN; L2 = UUR; S1 = AGN; and S2 = UCN. A solid line indicates a Watson-Crick bond, whereas a circle indicates a bond between G and uracil. All 22 of these tRNA genes were located in the same relative position and orientation within the mitochondrial genomes of both *Demodex* species. Furthermore, orthologous tRNA structures all looked similar between *Demodex* species; to see this, compare each tRNA in panel **(a)** versus panel **(b)**. Most of the tRNA molecules lacked at least one of the side arms, and some lacked both, suggesting that *Demodex* must have the molecular machinery necessary for these extremely truncated tRNAs to function.

We identified models for the standard suite of 22 tRNA genes within the mitochondrial genomes of each *Demodex* species (Figure 4). Importantly, each of these putative tRNA genes was located in the same relative position and orientation within the mitochondrial genome in both species. Furthermore, orthologous tRNA sequences could be aligned reasonably well between the two *Demodex* species. Finally, for all of the putative genes, the inferred tRNA secondary structures looked similar in both species; this can be seen by comparing the inferred structures from *D. brevis* (Figure 4a) and *D. folliculorum* (Figure 4b) for each orthologous pair of tRNA genes. Taken together, these observations provide substantial support for these gene models. If these were not tRNA genes, then it would seem extremely unlikely that the same relative stretch of DNA in both species would happen to have the potential to serve as the template for the same type of tRNA molecule, to exhibit substantial sequence similarity, and to have similar inferred secondary structures. We concluded that these gene models represent a plausible set of tRNA genes for both species, despite the fact that most of them possessed unusual structures.

Interestingly, most of the *Demodex* tRNA genes were truncated, lacking the sequence necessary to encode arms of the canonical cloverleaf-shaped tRNA (Figure 4). Eight pairs of orthologous tRNAs had just D-arms; three had just T-arms; and five had neither D- nor T-arms. In contrast, just six pairs of orthologous tRNAs retained both D- and T-arms and could fold into the canonical cloverleaf-shaped tRNA. Because the same basic structure could be inferred for each gene in both *Demodex* species, we concluded that these tRNA structures were probably present in the common ancestor of these lineages. The average sizes of tRNA genes in *D. brevis* and *D. folliculorum* agreed closely with each other: 53.5 bp (SE 1.4 bp) and 53.3 bp (SE 1.2 bp), respectively. These collections of tRNA genes were about the same size as the average value of 54.8 bp (SE 1.0 bp) reported previously for the tRNAs within mitochondrial genomes of Acariformes [19]. It appears that truncated mitochondrial tRNA genes were already widespread in the common ancestor of the Acariformes.

Among metazoans, more than 90% of mitochondrial tRNAs are inferred to share the common cloverleaf-shaped secondary structure of nuclear-encoded tRNA sequences [30]. Nevertheless, tRNAs that have lost one arm—either a D-arm or a T-arm—are extremely common among Acariformes [14–19]. Indeed, numerous examples of mitochondrial tRNAs that have lost one arm or the other can be found in several additional lineages of the chelicerates [28, 31]. Furthermore, it is common for metazoan mitochondrial genomes generally to possess one to a few tRNA genes that lack either D-arm or T-arm sequence [32]. These noncanonical tRNAs are thought to remain functional due to coevolution of interacting factors. For example, seryl-tRNA synthetase has evolved to be able to recognize the noncanonical tRNA-Ser in mammalian mitochondria [33]. In nematodes, one paralog of EF-Tu has evolved to bind tRNAs that lack the T-arm [34]. Presumably, similar forms of coevolution have occurred in order for the many truncated mitochondrial tRNAs of Acariformes, including *Demodex*, to remain functional.

Mitochondrial tRNA genes that have lost both D- and T-arms—resulting in minimal tRNAs with only acceptor and anticodon arms—are extremely unusual; nevertheless, these “armless” tRNAs have been inferred in some mitochondrial genomes previously [30]. Indeed, another member of the Tetranychidae (*Panonychus ulmi*) apparently has at least one such armless tRNA [19]. The trend towards the loss of these tRNA arms appears to have continued in *Demodex*, resulting in several examples of tRNAs that lack both D- and T-arms. Interestingly, a recent study used RT-PCR and 5′- and 3′-RACE to show that several of these miniaturized, armless tRNAs are indeed transcribed and correctly processed by CCA addition, at least in one species of nematode [35]. If these are actually not functional tRNA genes, then the functional copies must have been imported into the nuclear genome, and we are left with the puzzle of what could be maintaining these sequences.

The similar sequences and conservation of apparent structures for these putative tRNA genes, despite the fact that these are not closely related species, suggests that selection is maintaining these structures. If the truncated tRNA genes are actually no longer functional, then one hypothesis is that these sequences actually function as promoters, and just happen to fold into a remnant of a tRNA gene in both species. This seems implausible, however, given the high levels of divergence often seen in mitochondrial promoter sequences (e.g., [36]), which would lead us to expect the loss of anything resembling a folded tRNA.

Many of the inferred tRNA structures in both species had acceptor arms with fewer than seven paired bases (Figure 4). Indeed, the number of inferred structures with the full complement of seven paired bases in the acceptor arm was only six in *D. brevis* and 11 in *D. folliculorum.* To be functional, these molecules with truncated acceptor arms would presumably require either coevolved interacting molecules or RNA editing. Post-transcriptional editing of minimal tRNA molecules, including extensive modification of the acceptor arm, has been demonstrated in velvet worms [37].

The parallel evolution of truncated mitochondrial tRNA genes among chelicerates suggests that this group has evolved a novel mechanism that either permits these tRNAs to function in a truncated state and/or allows post-transcriptional editing to repair them. Determining the mechanisms responsible for keeping such minimal tRNA genes functional in *Demodex* will be an interesting subject of future research. These results could have implications for developing treatments that affect *Demodex* without harming the human host, since it has the potential to provide an avenue for the development of new acaricides that target the molecular machinery necessary for the function of such highly unusual tRNA genes.

### Phylogenetic position and species divergence

**Figure 5:**
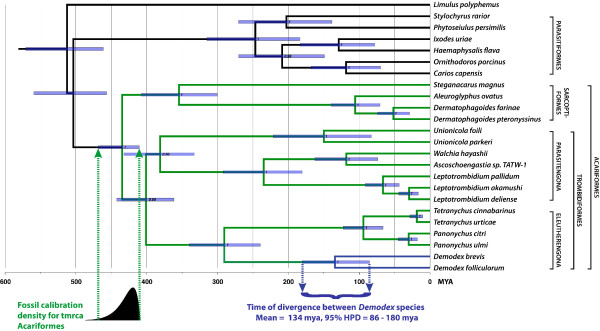
**Phylogenetic analysis using a relaxed molecular clock based on mitochondrial protein sequences to estimate the age of divergence between** ***Demodex*** **species.** Traditional groupings within the Acari were recovered in all phylogenies constructed. This phylogeny represents the results for one particular fossil calibration density, which is based on the minimum age of 410 mya for the time to the most recent common ancestor of the Acariformes (as inferred based on the four oldest Acariforme fossils identified in [39]); the particular fossil calibration density used in this analysis is depicted at the bottom of the tree, along with dashed arrow pointing to the common ancestor of the Acariformes. Amino acid sequences were used for all age-estimation analyses to minimize the effects of mutation saturation. For the particular analysis depicted in this phylogeny, the resulting estimated mean time to the most recent common ancestor of the two *Demodex* lineages was 134 mya, with a 95% highest probability density interval of 86 – 180 mya. Similar age estimates were obtained for two other fossil calibration densities, one that placed greater probability density near 410 mya, and one with a uniform distribution between 410–510 mya (Table 1). Furthermore, these estimates were not altered substantially when two species of Solifugae were included in the analysis (Table 1 and Additional file 1: Figure S1). Finally, these results are broadly overlapping with the results obtained based on an alignment of 18S sequences. Overall, these results suggest that *Demodex* have probably been coevolving with their mammalian hosts since before the placental radiation.

Our phylogenetic analysis recovered traditional groupings within the Acari (Figure 5). For example, the maximum clade credibility tree recovered the Acariformes as monophyletic, with the expected deep split into Trombidiformes and Sarcoptiformes. Within the Trombidiformes, the Eleutherengona and Parasitengona were recovered as monophyletic groups. The posterior probabilities for most of the clades were high. When the Solifugae were included in the analysis, the *Unionicola* actually fell outside of a clade that included the Eleutherengona plus five members of the Trombiculidae (Additional file 1: Figure S1). Otherwise, tree topologies were completely stable across analyses, regardless of whether the Solifugae were included or not. *Demodex* always clustered with the Tetranychidae—the spider mites—as expected, since both are considered members of the Eleutherengona (Figure 5). This result agrees with both traditional taxonomy [38] and a recent phylogenetic analysis based on nuclear rRNA sequences [11].

In the absence of either a fossil record or estimates of molecular divergence, it is unclear how long *Demodex* have inhabited the mammalian skin. For example, it is possible that *Demodex* were present on the mammalian common ancestor, and have been speciating with their hosts ever since. Since we do not have estimates of how often these mites can move between host lineages, however, it is also possible that *Demodex* evolved to parasitize mammals much more recently and are widespread across mammalian lineages simply because they have repeatedly colonized new host species.

Interestingly, the results of the relaxed molecular clock analysis based on the amino acid sequences of mitochondrial proteins suggest that the split between *D. brevis* and *D. folliculorum* happened a very long time ago (Figure 5 and Table 1). For example, the youngest average estimated time of divergence between these two *Demodex* lineages was 129 mya, with a 95% highest posterior density (HPD) interval of 87 – 173 mya. Across six different relaxed molecular clock analyses, the average lower bound of the 95% HPD interval was 87 mya for the time of the most recent common ancestor of the two *Demodex* lineages. Assuming that the transition to living on mammals happened once, these results suggest that the genus *Demodex* has been evolving and diversifying with their mammalian hosts for at least this long.

**Table 1:** **Summary statistics of relaxed molecular clock estimates of the divergence time between** ***Demodex*** **species based on amino acid sequence alignments of mitochondrial proteins**

Prior on tmrca [Acariformes]	Solifugae included?	tmrca [Acari] (mya)	tmrca [ ***Demodex***] (mya)
Exponential [10] Offset 410	No	483 [450 – 523]	129 [87 – 173]
Gamma [2,15] Offset 410	No	504 [457 – 560]	134 [86 – 180]
Uniform [410, 510]	No	520 [456 – 589]	137 [88 – 186]
Exponential [10] Offset 410	Yes	475 [441 – 509]	133 [82 – 185]
Gamma [2,15] Offset 410	Yes	494 [448 – 546]	140 [88 – 191]
Uniform [410, 510]	Yes	509 [449 – 578]	145 [93 – 197]

mya = millions of years ago; tmrca = time to most recent common ancestor. tmrca[Acari] represents the estimated divergence time between Acariformes and Parasitiformes. tmrca[*Demodex*] represents the estimated divergence time between *D. brevis* and *D. folliculorum.* Shown for the tmrca estimates are the mean and 95% highest probability density intervals for each analysis.

The average age estimate across analyses for the *D. brevis – D. folliculorum* split was 136 mya (Table 1). For comparison, these same analyses resulted in average estimated times to the most recent common ancestor of the Acari of 483 – 520 mya. It is remarkable that the split between *D. brevis* and *D. folliculorum* appears to have happened about 26 – 28% as long ago as the original divergence between Acariformes and Parasitiformes within the Acari, especially given that these *Demodex* species are so similar morphologically [1].

To calibrate our relaxed molecular clock analyses, we used a date of ~410 mya as the minimum age of the Acariformes, based on the four oldest acariform fossils so far identified [39]. These fossils include three that are considered members of the Endeostigmata, and one that is considered a member of the Trombidiformes. Molecular phylogenies support the hypothesis that the Endeostigmata represent a basal lineage of the Sarcoptiformes [40]. If, however, the endeostigmatan fossils actually turn out to represent a basal lineage of the Acariformes (i.e., falling outside of the Sarcoptiformes-Trombidiformes clade), then our fossil calibration date of 410 mya would not accurately represent the common ancestor of the Sarcoptiformes and Trombidiformes, which would bias the estimated age of the *D. brevis* – *D. folliculorum* split, making this split appear older than it is in reality.

To test the impact of different fossil calibration densities on our age estimates, we used three different distribution densities for the priors on age of the Acariformes (Table 1), all based on the ~410 mya time of appearance of members of this group in the fossil record [39]. Prior distributions used to represent the age of the Acariformes varied from highly restrictive (i.e., using an exponential distribution with most of the density near the 410 mya minimum age), to much less informative (i.e., using a uniform distribution with a range from 410 – 510 mya). Altering the prior distributions had no substantial effect on the estimated age of the *Demodex* divergence.

To test the impact of different taxon combinations on our age estimates, we varied the relaxed molecular clock analysis to either include or exclude two members of the Solifugae (Table 1). These taxa were chosen because two recent studies place them as the sister group to the Acariformes, which would make the Acari a paraphyletic grouping [40, 41]. We did not observe any sensitivity of the *Demodex* age estimates to whether Solifugae were included in the analysis. Furthermore, we did not see any evidence that the Solifugae are the sister taxon to Acariformes; in other words, when the Solifugae were included in the analyses, we always recovered a monophyletic Acari, with the posterior probability of the node uniting the Parasitiformes and Acariformes into the Acari always equal to one, providing strong support for the traditional grouping (to the exclusion of the Solifugae). Although this was by no means a comprehensive test of the higher order relationships among the Chelicerata, these results should nevertheless be added into the growing debate as to the correct topology of these relationships.

The common ancestor of the placental mammals has been estimated to have lived 72 – 108 mya, with the radiation of placental ordinal level crown groups occurring later [42]. Hence, our molecular clock results indicate that the two species found on humans may have last shared a common ancestor prior to the ordinal level radiation of the placental mammals, which is consistent with an ancient colonization of mammals by the *Demodex* lineage. Such an ancient colonization event would help to explain the wide distribution of *Demodex* across mammalian orders, as well as the clear morphological adaptations to living in the pilosebaceous complex. Furthermore, since Jurassic mammals had fur, these data are in agreement with the fossil record of mammals [43].

One potential shortcoming of our dating analyses is that mutation saturation could be influencing the results. In particular, if there is substantial saturation of the substitutions among the oldest splits in the phylogeny, then this will tend to cause the over-estimation of split ages near the tips of the tree, which could make our estimation of the *D. brevis – D. folliculorum* split look older than it actually is. For this reason, we used amino acid sequences rather than DNA sequences for our molecular dating analyses. Owing to their reduced state space (four possible bases), nucleotide sequences will saturate much more rapidly than protein sequences (20 possible amino acids). Furthermore, to assess whether there was substantial saturation among the amino acid substitutions, we also used the software AsaturA [44] to visualize the accumulation of amino acid substitutions with genetic distance, which showed that there was only very slight saturation of even the most frequent types of amino acid substitutions in these data. This suggests that our estimate of an ancient split between *D. brevis* and *D. folliculorum* was not being caused artificially by mutation saturation.

A second potential shortcoming of our dating analyses is that they are based on a single fossil calibration point. If there have been substantial changes over time in the rates of molecular evolution of the mitochondrial protein-coding genes used in the analysis, then this will tend to reduce the accuracy of our estimated *D. brevis – D. folliculorum* divergence time. On the other hand, the analysis method modeled evolution in each branch of the phylogeny with a relaxed molecular clock, meaning that rates of change were allowed to vary, which should have helped to ameliorate problems associated with slowdown or speedup of the rates. Furthermore, the age estimate is based on aligned sequences of four different proteins, each of which was allowed to slow down or speed up independently in the analysis. Finally, inspection of the rates estimated for each gene along different branches of the phylogeny did not indicate that there was a systematic change in evolutionary rates in the lineage leading to the *Demodex* species within the Acariformes. Nevertheless, because we based our divergence time estimates on a single fossil calibration date, the possibility remains that undetected changes in molecular evolutionary rates influenced the results.

One way to address these possible shortcomings is to ask whether the divergence estimate based on another gene is consistent with those based on the mitochondrial protein sequences. To this end, we conducted a relaxed molecular clock analysis based on a collection of 18S rDNA sequences for the Acariformes, with *L. polyphemus* as an outgroup. Based on the 18S alignment, the mean time to most recent common ancestor of *D. brevis* and *D. folliculorum* was 74 mya, with a 95% HPD interval of 12 – 150 mya. Unfortunately, this is a large time interval, which does not provide a strong test of our original age estimates. Nevertheless, this interval is broadly overlapping with the results of our analyses based on the mitochondrial protein sequences (i.e., compare this result to the intervals reported in Table 1), so our original estimates based on mitochondrial proteins are not contradicted by this estimate using 18S rDNA sequences. Furthermore, the average estimate of 74 mya based on the 18S genes would still make this an ancient split between *D. brevis* and *D. folliculorum* (i.e., still occurring prior to the radiation of placental mammals). If the area of overlap between the 95% HPD intervals based on both 18S genes and mitochondrial proteins is considered, then we would estimate that these taxa split somewhere between about 87 – 150 mya.

*D. brevis* and *D. folliculorum* live in distinct habitats within the skin: the former inhabits the sebaceous glands whereas the latter resides in the hair follicles nearer the skin surface, alongside the hair shaft. The ancient divergence observed between these lineages is consistent with the hypothesis that there was an early separation into mite lineages that specialize in either the hair follicle or the sebaceous gland. This hypothesis has been suggested previously [45], based on the observation that there are consistent morphological differences among mites from different skin microhabitats—mite species from different hosts but found in the same microhabitat tend to display more similarities than those living on the same host but in different microhabitats. These similarities are not restricted to overall shape, as might be expected to result from natural selection for squeezing into particular skin structures, but have also been observed in morphological characters that are regarded as diagnostic in taxonomy of Demodicidae. This hypothesis could be tested by comparing sequence divergence among additional *Demodex* species that reside either deep in glands within the skin versus near the surface of the follicle in other mammalian species. If correct, this hypothesis predicts that gland-dwelling versus follicle-dwelling species will tend to group into distinct clades. Although a small amount of sequence information is presently available for several other members of the genus, the classification of these species into primarily follicle- versus gland-dwelling taxa is unfortunately not clear, unlike the case for *D. brevis* versus *D. folliculorum*. A further test of this hypothesis will need to await further information about the primary habitats for taxa with available sequence information*.*

## Conclusions

These represent the first determinations of the complete mitochondrial genome sequences from any member of the genus *Demodex*. The availability of this additional genetic information promises to open the way for studies of variation within and between *Demodex* species. This is important because there has apparently been an ancient radiation of these mites on their mammalian hosts. Furthermore, *Demodex* are known to be ubiquitous in humans, and to cause medically important skin disorders.

We found that *Demodex* mitochondrial genomes share the following features with other members of the Acariformes: (1) they are compact in size and AT-rich; (2) they have experienced multiple gene rearrangements, especially among the tRNA genes; and (3) they have many truncated tRNA genes.

It remains unclear what is driving the evolution of truncated tRNA genes. Perhaps these are favored simply because of their reduced size, resulting in less RNA needing to be transcribed in order for translation to occur, and also less mitochondrial genome to be replicated. This hypothesis does not explain why selection for smaller tRNA genes has been so much more effective in certain lineages, such as the Acariformes. To be functional, the many truncated tRNA genes found in the mitochondrial genome would seem to require some combination of coevolved interacting factors and/or extensive tRNA editing. The molecular machinery necessary for dealing with truncated tRNAs might provide avenues to treat the apparently harmful *Demodex* found in some patients.

Based on the four most slowly evolving protein-coding genes within the mitochondrial genomes of the Acari, we examined the phylogenetics of the Acariformes, and supported the traditional hypothesis that the *Demodex* are members of the Eleutherengona. Interestingly, we found that the two *Demodex* species found on humans apparently diverged more than 87 mya, which is prior to the estimated time of the radiation of the placental mammals. Assuming a single transition to living in mammalian skin, this estimate places a lower bound on how long *Demodex* have been living on their mammalian hosts. Furthermore, the deep divergence time estimated between these two species is consistent with the hypothesis that there was an early split into distinct forms that either live deep in the sebaceous glands or near the surface alongside the hair shaft. This niche-separation hypothesis can be tested by examining the phylogenetics of additional *Demodex* species that have been shown to live in different skin microhabitats.

## Methods

### Ethics statement

Prior to sampling, each participant to be sampled for *Demodex* was informed both verbally and in writing about the goals of the project and the sampling protocol. All participants signed a written Informed Consent form. All human *Demodex* sampling procedures and the Informed Consent form were approved by Bowdoin College’s Research Oversight Committee, Approval No. 2007–34.

### Mite isolation and molecular techniques

Mites were isolated by drawing the curved end of a new bobby pin across the forehead of each participant. The resulting exudate was searched for mites in mineral oil under a stereomicroscope. Individual mites were verified as exhibiting the genital morphology of either *D. folliculorum* or a *D. brevis* (per [1]) using 600x magnification on a compound light microscope. To purify DNA, each mite was washed several times in fresh mineral oil, then the mineral oil was removed by washing 10x with 100% ethanol; the ethanol was evaporated by heating 2 min at 95°C, and the dried mite was then resuspended in 10 μL Lysis Buffer (10 μL PCR Buffer + 8 units Proteinase K in 10 μL H_2_O + 80 μL 1% Triton X), incubated 60 min at 65°C followed by 10 min at 95°C, then frozen for at least 1 hr. For initial PCR experiments the DNA prepared from multiple mites was pooled, whereas DNA from a single female mite from each species that had been diluted 100-fold in water was used as template for the PCR experiments necessary to generate the final mitochondrial sequences.

The first fragment successfully amplified from *D. folliculorum* was a fragment of the 12S rRNA gene, which was obtained using degenerate PCR based on an alignment of 12S rRNA from various arthropods. Primers were then designed within this small fragment so that long-range PCR could be used to amplify the rest of the mitochondrial genome. The resulting PCR product was ~14 kb in length, and the sequence of that large product was determined with a combination of primer walking and subcloning of fragments into plasmids using various restriction enzyme digestions and ligations, followed by plasmid sequencing, together with PCR experiments designed to link subcloned fragments. Once a draft of the mitochondrial genome had been assembled, a collection of PCR products that covered the entire sequence was generated from a single mite; these products were sequenced on both strands to determine the final mitochondrial genome sequence.

The first fragment successfully amplified from *D. brevis* was part of the ND5 gene, using PCR primers designed from the *D. folliculorum* mitochondrial genome. Otherwise, the same techniques were employed as above to generate a draft of the *D. brevis* genome, and then to determine the mitochondrial genome sequence from a single mite.

To verify the species identity of our final genome sequences, 20 individual mites from each species were isolated and identified based on the genital morphology described by Desch and Nutting [1]. DNA was prepared for each mite separately, and PCR amplifications using species-specific primer pairs (Additional file 2: Table S1), designed based on each mitochondrial genome sequence, were carried out to test species identity. Successful PCR amplifications with species-specific primers correlated perfectly with identifications based on morphology (P < 0.0001, Fisher’s Exact Test). Finally, we verified that our sequences agreed with fragments of the 16S [12] and COX1 [3] genes that have already been determined for both species. We concluded that the mitochondrial genome sequences correspond to the correct species.

Additional file 2: Table S1 lists the PCR primer pairs, annealing temperatures, and product sizes for all of the amplifications that were necessary to determine the mitochondrial genome sequences of both species. Additional primers were designed, as needed, for linking plasmid subclones during the compilation of the initial genome sequences, and for sequencing PCR products and plasmids. All primers were purchased from Invitrogen (Carlsbad CA, USA). Routine PCR was performed using GoTaq Hot Start DNA Polymerase (Promega, Madison WI, USA) or Phusion Hot-Start High-Fidelity DNA Polymerase (New England Biolabs, Ipswich MA, USA). Long-range PCR was accomplished using the Hot-Start version of TaKaRa LaTaq DNA Polymerase (Clontech Laboratories, Mountain View CA, USA). Both PCR products and plasmids were analyzed on 1% agarose gels. PCR was carried out using standard techniques in 50-μL volumes using an MJ Research PTC-200 thermocycler (Bio-Rad, Hercules CA, USA). PCR products were purified with the QIAquick PCR Purification Kit and plasmid DNA was purified using the QIAprep Spin Miniprep Kit (Qiagen Inc, Valencia CA, USA). Long-range PCR products were digested and subcloned into pBluescript II SK+ vector (Stratagene, La Jolla CA, USA) for plasmid sequencing using standard techniques. PCR products and plasmids were sent to either Geneway Research (Hayward, California, USA) or Mount Desert Island Biological Laboratory DNA Sequencing Core (Salisbury Cove, Maine, USA) for Sanger sequencing.

### Sequence annotation and inferences of secondary structures

All sequencing results were compiled and aligned for the purposes of determining the mitochondrial genomes, and oligonucleotide primers for both PCR and sequencing were designed, using MacVector (version 10.6, North Carolina, USA). *D. brevis* and *D. folliculorum* protein-coding genes were mapped by aligning the new genome sequences against both translated and untranslated sequences from a collection of taxa within the Chelicerata (Additional file 3: Table S2). The beginnings and endings of these coding sequences were based on putative start and stop codons nearest the ends of the alignable sequences. Locations and orientations of rRNA genes were mapped by alignment with rRNA genes from the same collection of chelicerate taxa, although the beginnings and endings of these genes were simply based on the limits of neighboring genes and so must be considered extremely tentative. The tRNA genes were identified by first analyzing the mitochondial sequences with four separate software packages that have been designed to search for tRNA genes: ARWEN [46], DOGMA [47], tRNAscan-SE [48], and MITOS [49]. Where possible, these software packages were all run using the most relaxed possible settings for recognition of tRNA structures. The resulting potential tRNAs were also aligned with the orthologous tRNAs from the standard collection of chelicerate taxa in order to verify their identity. Finally, a small number of tRNA genes could only be identified manually, and these were also verified by alignment. tRNA genes that were in the same relative physical locations in both *Demodex* species, exhibited a high degree of sequence similarity between *Demodex* species, showed at least a moderate degree of sequence similarity with other chelicerate sequences, and could be folded into plausible secondary structures, were considered real. Inferred tRNA structures were drawn using VARNA [50] and imported into Illustrator CS5 (Adobe Systems, Carlsbad CA, USA) for final graphics production.

The putative control regions were inferred as likely to comprise the largest noncoding stretch in each genome, while verifying that each of these also had a higher-than-average AT% nucleotide composition, although these stretches were too divergent to be aligned reliably between species.

### Gene order comparisons

Gene order comparisons were made using the SPRING web server [51], which takes a sample of chromosome gene orders and orientations as its input and then computes a minimum series of reversals and/or block-interchanges necessary for transforming each chromosome into every other chromosome. Codified gene orders and orientations for the 13 protein-coding genes plus the two rRNA genes were input into web server for the following: (1) gene arrangement of the horseshoe crab *Limulus polyphemus*, representing the ancestral chelicerate gene arrangement; (2) fourteen of the Acariformes species with available mitochondrial genome sequences (*Leptotrombidium pallidum* was excluded from the analysis because this species has a duplication within the mitochondrial genome); and (3) both *D. folliculorum* and *D. brevis*. The same analysis was completed including tRNA genes, except that *Steganacarus magnus* was excluded in this case because one tRNA gene is missing from that genome. The SPRING web server also estimates a distance tree based on the minimum number of breakpoints necessary to explain the gene arrangements; we downloaded the topology in Newick format and generated a drawing of this tree in FigTree, which was exported to Illustrator for final graphics generation.

### Phylogenetics and divergence time estimation

We chose to focus our phylogenetic and molecular clock analysis on four protein-coding genes that exhibit the slowest rates of nonsynonymous substitution, at least among the Acari [19]—COX1, COX2, COX3, and CYTB. Based on the protein sequences of these four genes, we inferred the phylogenetic position and divergence time of *D. brevis* and *D. folliculorum* based on a concatenated multiple sequence alignment that was generated using the GUIDANCE webserver [52], and the resulting alignment was used to estimate phylogenetic relationships and divergence times using the Markov chain Monte Carlo search of parameter space implemented in BEAST (v. 1.7.4) [53].

We extracted from the NCBI database the sequences for the COX1, COX2, COX3, and CYTB genes from the complete mitochondrial genomes of 21 Acari, plus the two available species of Solifugae, plus the horseshoe crab (*Limulus polyphemus*) as an outgroup (Additional file 3: Table S2). All of the available Acariformes in the database were included in the analysis (15 altogether), whereas only a small subset of the available Parasitiformes taxa were included, although these were chosen in order to sample a wide diversity within this clade. The Solifugae were included because both Dabert et al. [40] and Pepato et al. [41] provide support for the hypothesis that the Acari are actually paraphyletic, suggesting that the Solifugae actually group with the Acariformes to the exclusion of the Parasitiformes. We conducted the same analyses separately with the Solifugae included or excluded in order to compare the results of different taxon combinations on the phylogenetic positions and estimated divergence time of the two *Demodex* species. All DNA sequences were translated into amino acid sequences to minimize the influence of mutation saturation on the results.

To generate a multiple sequence alignment that consisted of only reliably aligned amino acids, thereby minimizing possible artifacts introduced due to alignment error, we used the online web server GUIDANCE [52]. Each individual gene was aligned separately, and the separate gene alignments were combined into a concatenated alignment (totaling 1279 or 1293 amino acids for the alignment including or excluding the Solifugae, respectively). The GUIDANCE alignment analyses were conducted using the MAFFT algorithm and 100 bootstrap replicates, with variable amino acid position columns identified using the GUIDANCE method and then removed based on a column-score cutoff of <0.93. The overall GUIDANCE scores for the each gene was always above 0.94, so most of each gene was included in the final alignment. The alignment file was converted to NEXUS format to be used as input for the software BEAUTi (version 1.7.4), which was used to set the Bayesian analysis parameters and to generate the input file for BEAST [53].

All BEAST runs were conducted using a chain length of 100,000,000, lognormal relaxed clock (uncorrelated), Yule process speciation tree prior, and an uninformative ucld.mean prior (exponential, mean = 1). The substitution and clock models for the four genes were unlinked, whereas the tree models were linked. Two separate BEAST analyses were conducted for each set of conditions, and the results inspected to verify that they converged on very similar parameter values and identical tree topologies, although the results of just one run for each set of conditions is reported. To calibrate the molecular clock, we used three different fossil calibration densities as priors for the age of the Acariformes, all based on the ~410mya time of appearance of members of this group in the fossil record [39]: (1) Exponential [10] with offset 410—this is the most restrictive prior, with most of the density very close to the 410 mya minimum age based on the fossil record, and 95% of the distribution density falling between 410 – 440 mya; (2) Gamma [2, 15] with offset 410—this distribution is somewhat less restrictive, and has greater density concentrated near the estimated Acariformes age of ~435mya reported by Dabert et al. [40], with 95% of the distribution density falling between 414 – 494 mya; and (3) Uniform [410, 510]—this is a relatively uninformative prior stretching over the 100-my interval starting from the oldest known Acariformes fossils. This allowed us to determine the effects of assuming different fossil calibration densities on the estimated age of the *Demodex* species divergence. All other settings were left in the default state or set to the uniform distribution with an arbitrarily large range. Tracer (version 1.5, [54]) was used to verify the convergence of runs by discarding the first 10% of the samples as burn-in, and examining the effective sample size of all parameters—the results were considered reliable only when the effective sample size of all parameters was above 100—as well as by visual inspection of the trace graphs for good mixing. We summarized the trees using TreeAnnotator ver. 1.7.4 [53], with the first 10% of trees discarded as burn-in; samples from the posterior were summarized on the maximum credibility tree for both the probability of each node as well as the 95% HPD of node heights. Finally, FigTree ver. 1.4.0 was used for tree visualization. The scalable vector graphic tree output from FigTree was imported into Adobe Illustrator (CS5) for final graphics generation.

Finally, we conducted a similar analysis to estimate time of divergence based on 18S rDNA gene sequences of various Acariformes that are available in GenBank. We conducted an analysis of estimated divergence based on a collection of 18S sequences, including *D. brevis*, *D. folliculorum*, 11 other Acariformes from both Sarcoptiformes and Trombidiformes, and *Limulus polyphemus* as the outgroup (Additional file 4: Table S3). The 18S rDNA sequences were aligned using the GUIDANCE web server, and the resulting alignment was inspected visually to insure that the inserted gaps resulted in a reasonable alignment across these taxa. The alignment was used to conduct a relaxed molecular clock analysis in BEAST using the same fossil calibration point as was used previously (i.e., the tmrca of Acariformes was assumed to be at least 410 mya) and the intermediate level of restriction on the prior for the time to most recent common ancestor of the Acariformes (i.e., assuming the Gamma [2, 15] distribution with an offset of 410 mya); we also assumed a general-time-reversible model of nucleotide substitution, with site heterogeneity of four gamma categories plus invariant sites, a lognormal relaxed clock (uncorrelated), and speciation was assumed to follow a Yule Process. Other settings were left as defaults.

### Availability of supporting data

The new mitochondrial genome sequences reported in this article are available in the GenBank repository, accession numbers KM114225 and KM114226. The accession numbers for the other complete mitochondrial genomes used in the analyses are listed in Additional file 3: Table S2. The accession numbers for the 18S rDNA gnes used for comparison are listed in Additional file 4: Table S3.

## Electronic supplementary material

Additional file 1: Figure S1: Phylogenetic analysis including two species of Solifugae. This phylogeny represents the same type of analysis as represented in Figure 5, except that in this case two species of Solifugae are included. The fossil calibration density is based on the minimum age of 410 mya for the time to the most recent common ancestor of the Acariformes, and amino acid sequences were utilized to minimize the effects of mutation saturation. For this analysis, the resulting estimated mean time to the most recent common ancestor of the two *Demodex* lineages was 140 mya, with a 95% highest probability density interval of 88 – 191 mya. Similar age estimates were obtained for two other fossil calibration densities, and these estimates were not altered substantially whether or not the Solifugae were included in the analysis (see Table 1 and Figure 5). The Solifugae were always recovered as a clade outside of the Acari. (PNG 902 KB)

Additional file 2: Table S1: Primer pairs used in PCR experiments for determination of *Demodex* mitochondrial genome sequences. (DOCX 163 KB)

Additional file 3: Table S2: Alphabetical list of taxa used for alignments to determine mitochondrial annotations and to estimate divergence time. (DOCX 113 KB)

Additional file 4: Table S3: Alphabetical list of taxa used for estimation of divergence time based on 18S rRNA genes. (DOCX 83 KB)

## Abbreviations

A: Adenine
T: Thymine
T-arm: Pseudouridine-arm of a tRNA secondary structure
D-arm: Dihydrouridine-arm of a tRNA secondary structure
rRNA: Ribosomal RNA
tRNA: Transfer RNA
12S: Gene for small subunit ribosomal RNA
16S: Gene for large subunit ribosomal RNA
Atp6 and −8: ATPase subunit 6 and 8
CO1-3 or COX1-3: Cytochrome oxidase subunits I-III
Cytb: Cytochrome b
ND1-6 or NAD1-6 and ND4L: NADH dehydrogenase subunits 1–6 and subunit 4 L
A+T: Large AT-rich non-coding region/control region
Bp: Base pairs.
