# Supplementary material for: Complete mitochondrial genomes of the human follicle mites Demodex brevis and D. folliculorum: novel gene arrangement, truncated tRNA genes, and ancient divergence between species
Source: BMC Genomics. 2014 Dec 16;15(1):1124. doi: 10.1186/1471-2164-15-1124 (PMC4320518; doi:10.1186/1471-2164-15-1124)
Supplement: Supplementary file 2 — Additional file 2: Table S1: Primer pairs used in PCR experiments for determination of Demodex mitochondrial genome sequences. (DOCX 163 KB) [file 12864_2014_6923_MOESM2_ESM.docx]

**Supplementary Table 1. Primer pairs used in PCR experiments for determination of *Demodex* mitochondrial genome sequences.**

| **Primer Pair** | **PCR Product Information** |
| --- | --- |
| F: AGAGTGACGGGCGATRTGTRCA  R: WWRRAIHRGGATTAGATACCCT | Amplified initial fragment of DF 12S rRNA gene  Anneal: Step-down 54 🡪 51˚C; Product: ~520bp |
| F: CTCTTCCTTTTCTAAAAATCTCCTCAATTCCCAAAAC  R: TTGAAACTTGATTTTGTTGGCGGTTTAAAGG | Long-range PCR to amplify DF mtDNA based on initial 12S gene fragment  Anneal: 63˚C; Product: ~14kb |
| F: CAAATCCACAAAAAACCCCC  R: TTGGCTATGGCTGCTCCTACTC | Amplified initial fragment of DB ND5 gene  Anneal: Step-down 52 🡪 49˚C; Product: ~500bp |
| F: AACTGTCTAAGAGTAGAAAAAAGCAATCACCC  R: AGGATTTTTAAGTTTGTTTATTTTATGTGGTTTAGG | Long-range PCR to amplify DB mtDNA based on initial ND5 fragment.  Anneal: 68˚C; Product: ~14kb |
| F: ACTAACCACAAAGACATTGGAACA  R: GAAAGAGCTTCTGAAGTTTCGGTC | DF single-mite sequencing  Anneal: 68˚C; Product: 630bp |
| F: CCCTGGACACTCCGTAGATTTAAT  R: AGTGTTACGTCTAGGGAGGAGTTA | DF single-mite sequencing  Anneal: 60˚C; Product: 687bp |
| F: ATGAGCCCACCACATATTTACAGT  R: AGTTGGCATTAGGATTGAATGAAGA | DF single-mite sequencing  Anneal: 59˚C; Product: 696bp |
| F: ACATTTTATAGGACTAAACGGCCT  R: GTTTAGTCGTCCTGGGATAGAGTC | DF single-mite sequencing  Anneal: 58˚C; Product: 789bp |
| F: ACTGAACTTACTCCTACCCAGAAC  R: TGTTGGGTCTTATGTTGGATGAGT | DF single-mite sequencing  Anneal: 59˚C; Product: 776bp |
| F: CAATTTGACCCATCGAACTCCTTC  R: TGTAGGCTTGTAAGATTGCTACCA | DF single-mite sequencing  Anneal: 60˚C; Product: 598bp |
| F: AACCCCAAACTTACTTGTACCCTT  R: AGGGCAATAGTTTACAGTTCTGGT | DF single-mite sequencing  Anneal: 60˚C; Product: 744bp |
| F: TCAGTAGCCATTTACGGAAACTGA  R: GAATCCACATTCGAAGGGTGAATT | DF single-mite sequencing  Anneal: 60˚C; Product: 660bp |
| F: AGCTGTATGATATTGGCATTTCGT  R: CACGAGTTGATTGGGCCTAAATTT | DF single-mite sequencing  Anneal: 59˚C; Product: 769bp |
| F: ATGTAGCCTTCTCTTTGGGGAAAT  R: TAGAGGTATCGGAAGGTGTCTCTT | DF single-mite sequencing  Anneal: 60˚C; Product:579bp |
| F: AGAAATTTGGATTAGGGGAATTAACT  R: CAGCAGTTTCGGTTAAGGGATTAA | DF single-mite sequencing  Anneal: 57˚C; Product: 754bp |
| F: TCTCCTCAATTCCCAAAACTTTCA  R: TGTGGATTTGGTTTTAGTGGATTTGT | DF single-mite sequencing  Anneal: 58˚C; Product: 757bp |
| F: TATAGACCAAAAGCTCCAACCACA  R: CTGGTATTATGGGGTTGGTTGAGA | DF single-mite sequencing  Anneal: 60˚C; Product: 786bp |
| F: CGTAATGCACTCCCTTAAACCAAG  R: TTTTGGCTAATCGATTTGGGGATG | DF single-mite sequencing  Anneal: 60˚C; Product: 792bp |
| F: AAGAAGAAACAGGAGTAGGAGCAG  R: TATGGCTTTGTAAGAGGGGTTTCT | DF single-mite sequencing  Anneal: 60˚C; Product: 766bp |
| F: CCAACTTCATACCTACACAACCAC  R: TTTGTTTTGTCTGCGAAGTTCTGA | DF single-mite sequencing  Anneal: 59˚C; Product: 581bp |
| F: ACCACGCATAATTAAAACTCTCCG  R: TGTTTTGGTTATGGGGAATTGAGG | DF single-mite sequencing  Anneal: 59˚C; Product: 790bp |
| F: GCATATAAACCCCAGCTCTCAAAC  R: TTTGGGAGTAAAAGGAGTGCTTCT | DF single-mite sequencing  Anneal: 60˚C; Product: 735bp |
| F: TTCTTCCCCTTCCACGAACTAAAT  R: TTTAGTGCTTTTGAGTGCAGTTCC | DF single-mite sequencing  Anneal: 60˚C; Product: 778bp |
| F: CCCTCTGTGAATTATTTCAATCGCA  R: GAAAGTGGATGGAAAAGAAACGGT | DF single-mite sequencing  Anneal: 60˚C; Product: 746bp |
| F: TCCCACACATGAATATCAGGGTTT  R: ACGGTTCTTCGATGACTTGTTTTC | DF single-mite sequencing  Anneal: 60˚C; Product: 703bp |
| F: TATTTGCTTACGCTATCCTACGCT  R: TTGTTTAGCTGAAATAAGCCGCTC | DF single-mite sequencing  Anneal: 60˚C; Product: 623bp |
| F: CAGTACACCACGAATAACCACAAA  R: TTTGGGAGAGCGTAAAATTTTGGG | DF single-mite sequencing  Anneal: 59˚C; Product: 721bp |
| F: AATAAATTAGCAATCCCCATAAAGG  R: TGAGTGGAGTAATGGGTGCTTTTA | DF single-mite sequencing  Anneal: 56˚C; Product: 704bp |
| F: AAAATCAAAAGCCAACATCGAGGT  R: AGAGCGAAATTAGTTGAGATAACTCT | DF single-mite sequencing  Anneal: 58˚C; Product: 765bp |
| F: AAACACCACTCCCAAGCTACTAAT  R: TGAGGGAAGAGTTTGGAAGATGAA | DF single-mite sequencing  Anneal: 60˚C; Product: 794bp |
| F: GGCTTGTCTTCAAACTCCTGAATC  R: TGGTGGGATTCCTGACAATGATAA | DF single-mite sequencing  Anneal: 60˚C; Product: 666bp |
| F: ACTCTGTTCAATCTACTTCCTCACC  R: TGTTCCAATGTCTTTGTGGTTAGT | DF single-mite sequencing  Anneal: 59˚C; Product: 777bp |
| F: TTATCATTGTCAGGAATCCCACCA  R: TGGAAAATTTTGCCAGGTTGTCTT | DF single-mite sequencing  Anneal: 60˚C; Product: 742bp |
| F: CTCTTCGAAAGTCTAACCTTCCCT  R: ACTTGTTCTGGTTTAATTTTCGGGT | DB single-mite sequencing  Anneal: 60˚C; Product 799bp |
| F: TCATAATCTTCTCCCTCCACATCG  R: ATTGCGAATACTGCTCCTATGGAT | DB single-mite sequencing  Anneal: 60˚C; Product: 733bp |
| F: GCCACAATAGTAATCGCAATTCCA  R: TTGGTTCATGTTTTGTTGGAGAGG | DB single-mite sequencing  Anneal: 60˚C; Product: 784bp |
| F: CTACTACCCAGACACCAACAATGA  R: ATTCCGCATAATTCTGAGCATTGG | DB single-mite sequencing  Anneal: 60˚C; Product: 680bp |
| F: GCTTAATCACTACCAGAAACGACG  R: TTTGTTGGTGAGTGTTAGTGATGC | DB single-mite sequencing  Anneal: 60˚C; Product: 604bp |
| F: CATCCTAAACTTCAACCCAAACCC  R: TGATGATGTATGCCTGTAGGATGG | DB single-mite sequencing  Anneal: 60˚C; Product: 712bp |
| F: AACTTCATACCCCACCTTAATCCC  R: TTCTTGATGAAAGGAGGAGGATGG | DB single-mite sequencing  Anneal: 60˚C; Product: 723bp |
| F: TCCCTAAGTCCTGCAATAGACCTA  R: GAGGTTTTGGATGGGTTGAAGATG | DB single-mite sequencing  Anneal: 60˚C; Product: 791bp |
| F: CTTCAACATCTCCCCAACAAAACA  R: TCCTTATCTTGTTTCTTTCCCCGA | DB single-mite sequencing  Anneal: 59˚C; Product: 796bp |
| F: AGAGATGAAACATTTTAACTTGCA  R: CGGAAGATGCTTCTTGTAAACCTC | DB single-mite sequencing  Anneal: 55˚C; Product: 754bp |
| F: ACTTATAGATTCTAAATCCATCGTACT  R: TTTAAGCAGGGTTGGGTGATTTTG | DB single-mite sequencing  Anneal: 56˚C; Product: 789bp |
| F: GCCCCTCTGTAAAACCTTTAAACC  R: GGGTTTTACCTGTTGTGGTTCTTT | DB single-mite sequencing  Anneal: 60˚C; Product: 787bp |
| F: ACTCTTCAAACTCACAAATCCCCT  R: GATGAAGGTTTGTTTTGGTAGGCA | DB single-mite sequencing  Anneal: 60˚C; Product: 634bp |
| F: AAGGCATACCAAACAAAGAACCAC  R: GGTTGGGATGGTTTAGGGATTACT | DB single-mite sequencing  Anneal: 60˚C; Product: 709bp |
| F: TGAACCAAAGAAGAAACCGGAGTA  R: TTGTTGGTATTGGTGGATTTGTGG | DB single-mite sequencing  Anneal: 60˚C; Product: 767bp |
| F: ACCAAACCATCACTCAAAACAAAA  R: ATGTGATTTGAGGGTTATGGTTGC | DB single-mite sequencing  Anneal: 58˚C; Product: 652bp |
| F: CGCATAACCAAAACTCTACGAGTC  R: TTTGTTTTGGTGATGGGTAGTTGG | DB single-mite sequencing  Anneal: 60˚C; Product: 788bp |
| F: CCTCAATCCAAATCCCAACACAAA  R: GAAGCAGAATGAAGTCTTTGGACA | DB single-mite sequencing  Anneal: 59˚C; Product: 740bp |
| F: ACCAATGAACTAACCGTCCCAATA  R: TGGATTGTTGAGTTTGAGGCTTTG | DB single-mite sequencing  Anneal: 60˚C; Product: 730bp |
| F: CCAAAGCATCAAACCTTACCACAA  R: ATTTGTCCTCATGGGAGTACGTAG | DB single-mite sequencing  Anneal: 60˚C; Product: 795bp |
| F: CATTCAACCGGAGCTTCACTATTC  R: GTTCTTCAATTACCTGGGCTCCTA | DB single-mite sequencing  Anneal: 60˚C; Product: 784bp |
| F: CCAACCCACTATCAACACCAAATC  R: TTTTAGAGGGTGAGTCTGAGTTGG | DB single-mite sequencing  Anneal: 60˚C; Product: 646bp |
| F: CAAGAACATCCCTCCAAAACACAA  R: TGGTAATAAGCGGTTTGGTCCTAA | DB single-mite sequencing  Anneal: 60˚C; Product: 749bp |
| F: AAACTTTCTCCTTCTTGCTCAACC  R: AGGCTAAAGTGTTCTTAAAGACGA | DB single-mite sequencing  Anneal: 58˚C; Product: 779bp |
| F: ACCAACATCGAGGTAACAAACTCA  R: TTGTATAAGGCAGTGAGGATTGGT | DB single-mite sequencing  Anneal: 60˚C; Product: 798bp |
| F: TGAAGCTTAACCATCAGCCCTTAA  R: TGAGAGAGTTGTTGGGTATTTGGT | DB single-mite sequencing  Anneal: 60˚C; Product: 715bp |
| F: GGAATCTCCTCCAATTCCTGACTA  R: AGAATGCTTGAGTTGAGTAGGGTT | DB single-mite sequencing  Anneal: 59˚C; Product: 764bp |
| F: ACCCTCAACATCCAATCTCTAACA  R: GGTTGGTTGATATGATTCATCGCA | DB single-mite sequencing  Anneal: 59˚C; Product: 799bp |
| F: TAGTAGAAACAAGCCCATGACCAA  R: TAGGTCTATTGCAGGACTTAGGGA | DB sequence identity confirmation  Anneal: 60˚C; Product: 308bp |
| F: TCAGTAGCCATTTACGGAAACTGA  R: CGTGAAGTCCAGTTGTCATGAAAA | DF sequence identity confirmation  Anneal: 60˚C; Product 304bp |

Forward and reverse primers that were used together successfully in PCR experiments are listed, along with relevant information for each such pair: purpose of the experiment, annealing temperature used for amplification, and the resulting product size. DF = *D. folliculorum*; DB = *D. brevis*.
