# Supplementary material for: Complete mitochondrial genomes of the human follicle mites Demodex brevis and D. folliculorum: novel gene arrangement, truncated tRNA genes, and ancient divergence between species
Source: BMC Genomics. 2014 Dec 16;15(1):1124. doi: 10.1186/1471-2164-15-1124 (PMC4320518; doi:10.1186/1471-2164-15-1124)
Supplement: Supplementary file 3 — Additional file 3: Table S2: Alphabetical list of taxa used for alignments to determine mitochondrial annotations and to estimate divergence time. (DOCX 113 KB) [file 12864_2014_6923_MOESM3_ESM.docx]

**Supplementary Table 2. Alphabetical list of taxa used for alignments to determine mitochondrial annotations and to estimate divergence time.**

| **Species** | **Lineage** | **Genbank**  **Accession** |
| --- | --- | --- |
| *Aleuroglyphus ovatus* | Acariformes, Sarcoptiformes | NC_023778 |
| *Ascoschoengastia sp. TATW-1* | Acariformes, Trombidiformes | NC_010596 |
| *Carios capensis* | Parasitiformes, Ixodida | NC_005291 |
| *Demodex brevis* | Acariformes, Trombidiformes | This study |
| *Demodex folliculorum* | Acariformes, Trombidiformes | This study |
| *Dermatophagoides farinae* | Acariformes, Sarcoptiformes | NC_013184 |
| *Dermatophagoides pteronyssinus* | Acariformes, Sarcoptiformes | NC_012218 |
| *Haemaphysalis flava* | Parasitiformes, Ixodida | NC_005292 |
| *Eremobates cf. palpisetulosus* | Arachnida, Solifugae | NC_010779 |
| *Ixodes uriae* | Parasitiformes, Ixodida | NC_006078 |
| *Leptotrombidium akamushi* | Acariformes, Trombidiformes | NC_007601 |
| *Leptotrombidium deliense* | Acariformes, Trombidiformes | NC_007600 |
| *Leptotrombidium pallidum* | Acariformes, Trombidiformes | NC_007177 |
| *Limulus polyphemus* | Merostomata (outgroup) | NC_003057 |
| *Nothopuga sp. 1 LP-2008* | Arachnida, Solifugae | NC_009984 |
| *Ornithodoros porcinus* | Parasitiformes, Ixodida | NC_005820 |
| *Panonychus citri* | Acariformes, Trombidiformes | NC_014347 |
| *Panonychus ulmi* | Acariformes, Trombidiformes | NC_012571 |
| *Phytoseiulus persimilis* | Parasitiformes, Mesostigmata | NC_014049 |
| *Steganacarus magnus* | Acariformes, Sarcoptiformes | NC_011574 |
| *Stylochyrus rarior* | Parasitiformes, Mesostigmata | NC_013474 |
| *Tetranychus cinnabarinus* | Acariformes, Trombidiformes | NC_014399 |
| *Tetranychus urticae* | Acariformes, Trombidiformes | NC_010526 |
| *Unionicola foili* | Acariformes, Trombidiformes | NC_011036 |
| *Unionicola parkeri* | Acariformes, Trombidiformes | NC_014683 |
| *Walchia hayashii* | Acariformes, Trombidiformes | NC_010595 |
