# Supplementary material for: Complete mitochondrial genomes of the human follicle mites Demodex brevis and D. folliculorum: novel gene arrangement, truncated tRNA genes, and ancient divergence between species
Source: BMC Genomics. 2014 Dec 16;15(1):1124. doi: 10.1186/1471-2164-15-1124 (PMC4320518; doi:10.1186/1471-2164-15-1124)
Supplement: Supplementary file 4 — Additional file 4: Table S3: Alphabetical list of taxa used for estimation of divergence time based on 18S rRNA genes. (DOCX 83 KB) [file 12864_2014_6923_MOESM4_ESM.docx]

**Supplementary Table 3. Alphabetical list of taxa used for estimation of divergence time based on 18S rRNA genes.**

| **Species** | **Lineage** | **Genbank**  **Accession** |
| --- | --- | --- |
| *Aleuroglyphus ovatus* | Acariformes, Sarcoptiformes | JQ000109.1 |
| *Demodex brevis* | Acariformes, Trombidiformes | GU377178.1 |
| *Demodex folliculorum* | Acariformes, Trombidiformes | GU377177.1 |
| *Dermatophagoides farinae* | Acariformes, Sarcoptiformes | JQ000247.1 |
| *Dermatophagoides pteronyssinus* | Acariformes, Sarcoptiformes | JQ000249.1 |
| *Leptus sp. AP-2010* | Acariformes, Trombidiformes | HM070355.1 |
| *Limulus polyphemus* | Merostomata (outgroup) | L81949.1 |
| *Microtrombidium sp. AP-2010* | Acariformes, Trombidiformes | HM070352 |
| *Mideopsis roztoczensis* | Acariformes, Trombidiformes | JN018219.1 |
| *Smaridiidae sp. AP-2010* | Acariformes, Trombidiformes | HM070364.1 |
| *Sonotetranychus sp. AP-2010* | Acariformes, Trombidiformes | HM070371.1 |
| *Steganacarus magnus* | Acariformes, Sarcoptiformes | AF022040.1 |
| *Tetranychus sp. AP-2010* | Acariformes, Trombidiformes | HM070369.1 |
| *Trombiculidae sp. AP-2010* | Acariformes, Trombidiformes | HM070354.1 |
